# Current status, challenges, and prospects of artificial intelligence applications in wound repair theranostics

**DOI:** 10.7150/thno.105109

**Published:** 2025-01-02

**Authors:** Huazhen Liu, Wenbin Sun, Weihuang Cai, Kaidi Luo, Chunxiang Lu, Aoxiang Jin, Jiantao Zhang, Yuanyuan Liu

**Affiliations:** 1 School of Medicine, Shanghai University, Shanghai, 200444, People's Republic of China.; 2 School of Mechatronic Engineering and Automation, Shanghai University, Shanghai, People's Republic of China.

**Keywords:** Skin injuries, Artificial intelligence, Deep learning, Wound diagnosis, Wound repair

## Abstract

Skin injuries caused by physical, pathological, and chemical factors not only compromise appearance and barrier function but can also lead to life-threatening microbial infections, posing significant challenges for patients and healthcare systems. Artificial intelligence (AI) technology has demonstrated substantial advantages in processing and analyzing image information. Recently, AI-based methods and algorithms, including machine learning, deep learning, and neural networks, have been extensively explored in wound care and research, providing effective clinical decision support for wound diagnosis, treatment, prognosis, and rehabilitation. However, challenges remain in achieving a closed-loop care system for the comprehensive application of AI in wound management, encompassing wound diagnosis, monitoring, and treatment. This review comprehensively summarizes recent advancements in AI applications in wound repair. Specifically, it discusses AI's role in injury type classification, wound measurement (including area and depth), wound tissue type classification, wound monitoring and prediction, and personalized treatment. Additionally, the review addresses the challenges and limitations AI faces in wound management. Finally, recommendations for the application of AI in wound repair are proposed, along with an outlook on future research directions, aiming to provide scientific evidence and technological support for further advancements in AI-driven wound repair theranostics.

## Introduction

As the body's largest organ, the skin is critical in protecting internal organs and tissues from external injuries, resisting foreign microorganisms, regulating temperature, and participating in immune responses 1-3. Injuries caused by physical, chemical, and disease-related factors can not only disrupt skin appearance and barrier function but also involve micro-level damage to local blood vessels and cells 4. In the United States, over 11 million people are affected by acute wounds annually, and more than 6 million suffer from chronic wounds, resulting in wound care costs exceeding $30 billion per year 5, 6. Severe skin injuries can lead to infection, amputation, systemic complications, and even life-threatening, posing a significant burden on healthcare systems and causing substantial economic and psychological stress on society 7, 8. Therefore, promoting wound repair and enhancing healing quality is clinically significant for reducing disability and mortality, especially in minimizing scar formation.

Current wound treatment methods, including dressing changes, skin grafting, artificial dermis, wound dressings, negative pressure therapy, and the administration of growth factors and cytokines, are limited by low overall efficacy, single functionality, and suboptimal outcomes 9, 10. These approaches also face numerous challenges, such as poor healing of chronic wounds, high infection risk, lack of real-time and accurate assessment, insufficient personalized care, limited medical resources, and significant economic burdens. Wound repair involves multiple interconnected stages, including hemostasis, inflammation, angiogenesis, dermal tissue regeneration, and remodeling. Due to the complexity and dynamic nature of wound healing processes, predicting the precise healing trajectory and effective clinical interventions remains challenging 11-13. Accurate wound diagnosis, including injury type and severity, aids in monitoring and analyzing wound conditions, and informing clinical decisions. Building upon this foundation, personalized treatment strategies that harness the body's self-repair capabilities can effectively promote wound healing and skin regeneration.

Artificial intelligence (AI) is a cutting-edge technological science used to simulate, extend, and enhance human intelligence 14. Currently, various AI technologies have been developed, including machine learning (ML), neural networks (NN), deep learning (DL), support vector machine (SVM), and electronic health records (EHR). AI excels in analyzing, presenting, understanding, and interpreting complex medical data, fundamentally transforming and reshaping global healthcare systems 15, 16. For example, AI can integrate medical images such as magnetic resonance imaging, CT scans, and 3D ultrasound reconstructions for automated diagnosis, significantly improving diagnostic efficiency and accuracy while optimizing healthcare resources. Moreover, AI techniques can efficiently process large amounts of unstructured data, extract crucial data, learn iteratively to accurately identify complex conditions, and provide recommendations for personalized treatment 17, 18.

**Figure 1:**
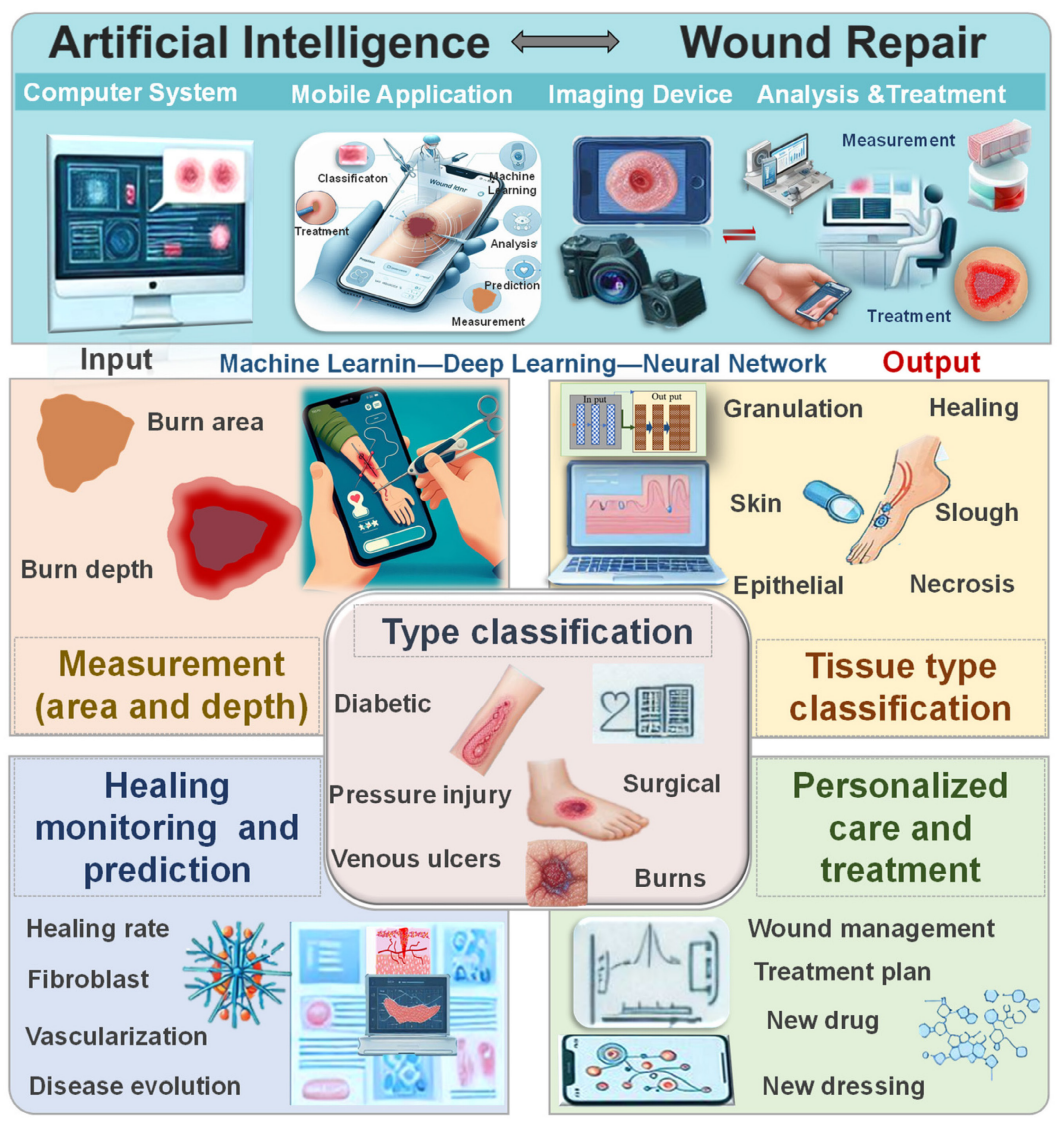
Applications of AI in wound repair theranostics include wound classification, measurement of wound area and depth, monitoring and prediction of wound healing, and personalized care and treatment strategies.

Traditional wound diagnosis and treatment require skilled and experienced clinical physicians, and the process can be time-consuming with the possibility of diagnostic errors. Currently, AI can leverage large datasets, including various textual and image data, to efficiently assist physicians in wound diagnosis 19. Subsequently, it aids in formulating treatment plans and optimizing wound management, leading to improved treatment outcomes and patient prognosis 20. AI not only supports clinical doctors but also provides less experienced training for nursing teams with limited skills, enabling remote healthcare and enhancing overall service quality. While AI-based methods offer decision support for wound care, the current focus is mainly on diagnosis and measurement techniques, with limited attention to treatment outcomes and strategies 19, 21. Challenges remain in achieving a closed-loop care system for the comprehensive application of AI in wound management, which should encompass wound diagnosis, monitoring, and treatment. Comprehensive reviews in this area are currently lacking. Hence, this review provides a comprehensive overview of recent advancements in AI applications in wound repair theranostics. It covers the current use of AI in injury type classification, wound area and depth measurement, wound tissue type classification, wound monitoring and prediction, and personalized treatment strategy development (Figure 1). Additionally, the article discusses the limitations and future potential of AI in wound management. Through in-depth analysis, this review not only demonstrates the practicality and efficiency of AI in optimizing wound diagnosis and treatment but also highlights the challenges and potential solutions. It provides scientific evidence and technological support to drive innovation and clinical practice in this field.

## AI diagnosis and wound type classification

Skin injuries can be classified based on various criteria, including cause, location, size, depth, exposure to the external environment, severity, healing time, and potential infection risk. Clinically, common wound types include diabetic foot ulcers (DFU), arterial ulcers (AU), lower extremity venous ulcers (VU), pressure ulcers (PU), and surgical wounds 22. Different types of injuries require different treatment approaches, making the development of high-precision classification models and accurate wound classification crucial for diagnosis, treatment planning, and prognosis. Researchers have proposed various innovative AI models to address this need. CNN in DL consists of convolutional layers, pooling layers, and fully connected layers, which can automatically extract local features of data, mainly focusing on extracting basic features such as edges and textures of images. For instance, as a novel convolutional neural network (CNN) architecture, DFUNet is often used to classify foot ulcers in diabetes. The framework combines the traditional convolution layer and parallel convolution layer to better capture feature differences in images and effectively identify different features between healthy skin and diabetes foot ulcers, such as edge, intensity, color changes, etc. Even on small datasets, DFUNet can accurately classify DFU and normal skin, with 10-fold cross-validation and an area under the curve (AUC) of 0.961 23. In addition, DFUNet can process input data more accurately and efficiently, reducing processing time. In addition, DFUNet can process input data more accurately and efficiently, reducing processing time. Machine learning algorithms can efficiently process massive amounts of data and automatically extract valuable features from the data, with accurate prediction and classification capabilities. Optimizing the error between predicted and observed results, helps computers learn all complex nonlinear interactions between variables, thereby achieving the goal of automatically optimizing the error between predicted and actual results. As a machine learning algorithm, SVM has the advantages of strong generalization ability, suitability for high-dimensional data, and insensitivity to data loss. Based on this, Wang *et al.* developed an SVM algorithm based on image color channels to determine chronic wound areas, which can analyze images on smartphones. This algorithm uses an SVM to determine wound boundaries and differentiate between healthy tissue and wound areas based on color and wavelet features, with an average sensitivity of 73.3% and specificity of 94.6% 24. As another branch of ML, DL can automatically extract features from raw data, with excellent complex perception and task understanding, and effective processing of high-dimensional data. It adapts to different tasks and dataset sizes by increasing the number of layers, and neurons, or changing the network architecture. Other studies have successfully used CNN to classify DFU and VU 25. Kim *et al.* 26 developed a high-precision classification model using ML algorithms, corresponding to existing injury severity scoring systems. This model can quickly and accurately triage patients at large-scale disaster sites via wearable devices, even in the absence of medical personnel. Sarp *et al.* used interpretable AI tools X-AI-cwc, transfer learning, and data augmentation techniques with the VGG16 network as the classification model, successfully categorizing chronic wounds into four types. The average F1 score was 0.76, with prediction accuracies of 95.36% for DFU, 100% for lymphatic injuries, 100% for pressure injuries, and 99.2% for surgical wounds 27. Another study developed an interpretable AI model using 2,957 images from the Singapore Advanced Institutes Image Registration Center, which analyzed vascular images in Asian populations and classified neuroischemic ulcers (NIU), surgical site infections, venous leg ulcers (VLU), and PU, achieving an average classification accuracy of 95.9% 28. Table 1 provides a summary of wound-type classifications.

Currently, hybrid models based on DL and transfer learning perform well in chronic wound classification and interpretation tasks. For example, SVM has the drawbacks of high computational complexity, sensitivity to parameters, and unsuitability for large-scale data. Transfer learning can achieve high accuracy with limited data and shorten training time by leveraging existing pre-trained models. The combination of transfer learning and DL can effectively address the shortcomings of SVM, enabling it to adapt to new task requirements more quickly, enhance the model's generalization ability, and reduce the risk of overfitting. AI technology overcomes the limitations of visual inspection by the human eye in distinguishing tissue types, and high-performance automated classifiers can assist medical personnel in accurately determining wound types. However, collecting more data and developing new methods, further improve feedback between AI and end users can guide clinicians and caregivers in making joint decisions, resulting in more accurate predictive insights and improved classification performance.

## AI-assisted wound measurement

The geometric shape and appearance of wounds contain important information about their cause, severity, duration, status changes, and healing expectations 29. Wound measurement (including size, area, or volume) is essential for diagnosis, treatment planning, and prognosis prediction, playing a crucial role in determining the healing trajectory of wounds. Monitoring the reduction in wound area or volume, and the growth of granulation/epithelial tissue, are key indicators of wound healing and treatment effectiveness 30. However, many clinicians still face challenges in ensuring consistency and accuracy in measurements. Traditional wound assessment relies on visual elements (such as erythema, granulation tissue (GT), and wound exudate evaluation) and accurate measurement methods using digital cameras, paper rulers, and depth probes to calculate wound area and volume 29, 31. AI has been widely applied in medical image segmentation due to its capabilities in denoising, contrast enhancement, and edge detection. Commonly used detection models include You Only Look Once (YOLO), single shot multibox detector, and regions of interest (ROI)-CNN 32; and popular classification models include YOLO, GoogLeNet, AlexNet, ResNet, and VGG 33. Studies have shown that the application of segmentation and outlier removal techniques can improve the classification accuracy of DL in distinguishing burn areas from surrounding healthy skin 34.

Assessing wound area and depth is fundamental to evaluating the extent of skin injuries and is critical for determining clinical treatment strategies. Currently, rapid and portable computer-aided diagnosis (CAD) tools and laser Doppler imaging provide automated assistance for burn assessment, but their high maintenance costs limit widespread use in hospitals 35. In contrast, DL imaging systems based on photography and spectroscopy offer more accessible solutions. Combined with DL algorithms (such as CNN and variational autoencoders), these systems can support clinicians in wound assessment and inform clinical treatment 36, 37. In summary, AI-enhanced wound measurement methods provide clinicians with more accurate and consistent assessment tools. This not only improves the accuracy of diagnosis and treatment but also better monitors the healing process, optimizing treatment strategies and enhancing patient outcomes.

### AI measurement of wound area

Accurate assessment of wound area is crucial for clinical treatment, particularly in burn cases where incorrect area evaluation can lead to improper fluid resuscitation, increasing the risks of fluid overload, shock, renal failure, and compartment syndrome 38. High-quality imaging is the basis of wound measurement 31. Digital wound measurement systems (DWMS) demonstrate high accuracy in challenging scenarios involving dark skin tones, indistinct wound edges, irregular shapes, unhealthy tissue, and NT 39. Three-dimensional measurement systems (3D-DWMS) have demonstrated reliability in two-dimensional area analysis, they have not yet met clinical standards for three-dimensional depth and volume analysis 40, 41. Sheng *et al.* 42 improved accuracy in burn area assessment by using three-dimensional reconstruction data combined with wound images, surpassing traditional geometric area calculation methods. As a type of DL, deep convolutional neural networks (DCNN) automatically learn object features in images and utilizes translation invariance and local perception characteristics to improve the effectiveness of local features, which is of great significance for complex burns. Based on this, researchers have developed a DCNN architecture for automatic wound and tissue segmentation, combined with diverse datasets, which can effectively overcome the limitations of traditional burn area assessment methods. A study proposed a DL-based method for burn wound detection, segmentation, and TBSA% calculation 43. Researchers trained U-Net and MASK R-CNN models using annotated burn wound images and healthy images. For burn wound image segmentation, the MASK R-CNN model combined with ResNet101 performed the best, achieving a Dice coefficient (DC) of 0.9496, while the U-Net combined with the ResNet101 model had a DC of 0.8545. Another study investigated the effectiveness of CNN trained on datasets for segmenting DFU and VLU. Under supervised learning, CNN based on egNet, LinkNet, U-Net, and Unet-VGG16 algorithms was trained using a dataset of sacral PU. The results showed that the CNN based on U-Net effectively segmented the wounds with the best performance and highest speed, achieving an AUC accuracy of 0.997, specificity of 0.943, and sensitivity of 0.993 44. Further research involved preprocessing input images to eliminate artifacts, followed by generating probability maps through CNN and finally extracting wound regions from the probability maps to address false positives 45.

The diversity of wound characteristics and the ambiguity of wound boundaries pose significant challenges in wound segmentation and assessment. To address this issue, Liu *et al.* 46 proposed a deep cross framework (WoundSeg) that includes data augmentation, segmentation networks, and post-processing to achieve automatic localization and segmentation of wound areas. This framework combines the complex feature extraction ability of DNN with the efficient polynomial relationship mining ability of cross networks to comprehensively understand data. Under five-fold cross-validation, WoundSeg achieved accuracy, sensitivity, precision, mean intersection over union (IoU), and DSC of 98%, 90%, 97%, 84.6%, and 91.66%, respectively. Additionally, a novel composite wound segmentation model combining traditional manual annotation with DL was proposed. This model integrates pre-background removal images with a deep neural network, achieving precise segmentation of wounds under semantic correction 47. With the excellent instance capability of MASK R-CNN, Munoz *et al.* achieved precise segmentation of images of DFU patients to evaluate wound healing during Heberprot-P treatment. By incorporating transfer learning, the proposed model's segmentation accuracy ranged from 93.90% to 98.01% 48. Jiao *et al.* combined a multi-scale CNN and a regional proposal network to improve the classification function of fully CNN instance segmentation, resulting in a burn wound segmentation framework 49. They evaluated three backbone networks: Residual Network-101 with Atrous Convolution in Feature Pyramid Network (R101FA), Residual Network-101 with Atrous Convolution (R101A), and Inception V2-Residual Network with Atrous Convolution, finding that R101FA had the highest accuracy (84.51%) and performed best in superficial, partial-thickness, and full-thickness burn segmentation. Recent studies have applied DCNN image segmentation algorithms, which involve inputting feature maps from standard convolution layers into convolution layers and mapping them to output feature maps via convolution kernels. Comparative analysis with traditional fuzzy C-means and regional convolutional neural network (RCNN) models showed that the DCNN model demonstrated higher accuracy in image segmentation tasks, significantly enhancing segmentation precision 50.

With the rapid development of AI technology and the widespread use of mobile devices such as smartphones and smartwatches, treatment methods in the healthcare field have become increasingly efficient. The integration of precise pattern recognition technology with cameras built into smartphones and tablets has significantly advanced the development of remote wound area measurement and assessment 31. A mobile device-based fully automated wound segmentation system, combining DL MobileNetsV2 with labeled datasets, achieved wound segmentation from natural images. This model features a simple framework and low computational requirements, enabling it to run on mobile devices, with a DC reaching 94% 51. Furthermore, researchers developed the AutoTrace model, a DCNN structure runnable on mobile devices, enabling precise and objective calculation and prediction of wounds and tissues, with average IoU reaching 0.8644 and 0.7192, respectively 52. To achieve high-precision data analysis with limited data, Rania *et al.* 53 evaluated the accuracy of three DL models (U-Net, V-Net, and Seg Net models) in segmenting DFU areas on mobile clients. The U-Net model performed well in this task, with a maximum accuracy of 94.96%, IoU of 94.86%, and DC of 97.25%. These studies indicate that mobile terminals are ideal media for wound image acquisition and data transmission, with broad application prospects 54. Additionally, a mobile device wound area measurement method relying on a multi-step process has been proposed, including steps such as image capture, grayscale conversion, blur processing, threshold segmentation, wound recognition, and expansion and erosion of wound areas, effectively enhancing measurement accuracy. By employing DL models supported by the OpenCV framework (such as U-Net, PSPNet, DeeplabV3+, and MASK R-CNN), this method demonstrates outstanding performance in tasks such as comprehensive wound segmentation, palm segmentation, and deep burn segmentation, with accuracies reaching 0.90767, 0.98987, and 0.90152, respectively 55. Using mobile applications such as Burn Med, CARES4WOUNDS (C4W), Swift, and Wound Aide for burn area prediction and wound size measurement has shown high reliability and accuracy information feedback between devices and users, significantly improving measurement speed and reducing evaluation time 56-60. Research has integrated FLIR™ infrared cameras with the Swift mobile application, providing skin temperature readings equivalent to clinical reference thermometers, enabling the Swift mobile application to have the advantage of a non-contact, user-friendly wound measurement tool. This allows clinical doctors to image, measure, and track wound size and temperature from one visit to the next, suitable for home monitoring by patients and their caregivers 61. For chronic wounds, Chan *et al.* 62 utilized the C4W mobile application to monitor the recovery process of DFU and compared it with traditional measurement methods to evaluate its measurement accuracy in terms of length, width, and area. The system showed reliability in measuring wound length, width, and area of DFU wounds in 8 patients, with reliability scores of 0.947, 0.923, and 0.965, respectively. Table 2 provides a summary of wound area measurement.

Although many current technologies primarily focus on wound segmentation to aid diagnosis, achieving a comprehensive evaluation still necessitates establishing labeled datasets for wound depth and developing dedicated assessment frameworks for more thorough wound analysis and management. One of the key challenges for future research is to enhance the versatility of models to adapt to a wider range of wound types and clinical scenarios, thus enabling more comprehensive and precise wound assessment. In conclusion, the application of DL and other advanced technologies in wound segmentation and analysis provides clinicians with accurate, rapid, and automated methods for wound assessment. The development and refinement of these technologies will provide stronger support for wound treatment, facilitate patient recovery, and improve medical efficiency. With ongoing technological advancements, the prospects for applications in remote healthcare and self-monitoring will be even broader in the future.

### AI measurement of wound depth

Currently, assessing wound depth is not only a crucial step in evaluating the severity of injuries but also forms the basis for devising clinical treatment plans. Inaccurate burn assessments can lead to improper wound management, often resulting in delayed healing or unnecessary surgeries 63. Differences in wound color and texture reflect varying degrees of dermal capillary damage and serve as the primary basis for diagnosing burn depth. Although clinical assessment remains a widely accepted method, the lack of timely and accurate early diagnosis can lead to inappropriate treatment and affect prognosis. Therefore, using image analysis technology to assist in diagnosing burn depth has significant theoretical and practical value 64.

Although a large number of AI algorithms have been developed and validated for wound image analysis, outliers can lead to a decrease in accuracy, especially when training models. The SVM and k-nearest neighbor (KNN) classification methods based on supervised learning algorithms can separate data points of different categories as much as possible to remove outliers. Based on this, Li *et al.* 65 trained multiple burn classification models using SVM and k-nearest neighbor (KNN) classification methods, and developed a multi-stage method based on Z-test and univariate analysis to improve the classification accuracy of deep injuries, wound beds, and partial injuries by removing outliers. Under 10-fold cross-validation, the accuracy reached 76%. In another study, researchers addressed the shortage of specific scene images by integrating transfer learning into a convolutional neural network-based ResNet50 model, achieving a diagnostic accuracy of 80% for three burn types (superficial, intermediate, and deep) 66. To address color factors in CAD image analysis, Acha *et al.* combined psychophysics with multidimensional scaling (MDS) analysis to enhance burn depth judgment. By leveraging its powerful visualization capabilities for complex high-dimensional data, MDS analysis outperforms SVM in terms of specificity and positive predictive values, reaching 0.94 and 0.94, respectively 64. Furthermore, AI burns diagnostic systems based on multimodalities such as ultrasound and RGB images have been developed, with texture features provided by ultrasound improving the accuracy of depth classifiers to 80% 67. The U-Net CNN is capable of integrating low-level detail features with high-level semantic features, enabling precise segmentation of target areas. Researchers combined this model with a high-performance polarized optical camera to accurately assess wound depth. The findings indicate that the accuracy of this research in diagnosing pediatric burns is nearly 97% 68.

Cirillo *et al.* combined DCNN with Tivi cameras for nontemporal evaluation and prediction of wounds. The ResNet-101 model achieved average, minimum, and maximum accuracies of 81.66%, 72.06%, and 88.06%, respectively, in 10-fold cross-validation. This demonstrates that CoHI outperforms deep CNN in wound recognition accuracy, achieving 90.54% accuracy, 74.35% sensitivity, and 94.25% specificity across different burn depths 35. In traditional wound image processing and analysis, multi-parameter and high-dimensional methods are often used to achieve high accuracy, which brings difficulties to processing complex images. The VGG-16 network based on DCNN can effectively extract local features of images while reducing the number of parameters. In addition, using pooling layers to maintain the main features of the image reduces data dimensionality, computation, and parameter count. Based on this, Despo *et al.* used an improved fully convolutional network (FCN) DL method based on the VGG-16 network for burn classification and developed an end-to-end DL model to evaluate specific burn wound features. The model was trained on a new dataset containing four types of wound images and achieved accurate classification. The binary classification accuracy was as high as 96.4%, with an average of 94.28%, and the ternary classification accuracy was as high as 91.9% and 87.7% 69. Table 3 provides a summary of wound depth measurement.

AI technology shows promise in automatically predicting burn depth, and integrating image acquisition with DL algorithms can significantly improve diagnostic accuracy. However, the current methods achieve an accuracy of only about 90%, indicating the need to expand datasets, select appropriate imaging modalities, and optimize algorithms to further enhance accuracy 70, 71. In summary, accurate assessment of burn depth remains a critical and challenging aspect of injury evaluation. Despite progress in burn depth detection, the irregular shapes and significant color variations of chronic wounds, along with the similar appearance of wounds with different depths and tissue compositions, suggest that these technologies alone may not be sufficient for precise evaluation. Automated and objective diagnostic methods are the ideal widely accepted approach, but the clinical experience and expertise of physicians are still required.

## AI diagnosis and wound tissue type classification

The composition of tissue types within a wound (epithelial tissue (ET), GT, slough, necrotic tissue (NT), and eschar) is a crucial indicator of the healing process. Accurate estimation of tissue components allows clinicians to select appropriate dressings, identify wounds at risk of non-healing, refer patients to specialists promptly, tailor treatment according to the patient's condition, and optimize wound care and healing outcomes. High-performance automatic classifiers can assist or augment clinicians in classifying wound tissue types, especially in resource-limited settings 72, 73. The application of AI in image recognition offers an effective solution for automatically classifying wound tissues 74. However, automatic wound classification is affected by environmental noise, heterogeneity, and inaccuracy in image capture. To this end, Veredas *et al.* 75 utilized a mean shift process combined with region-growing methods to effectively segment pressure ulcer regions and extract color and texture features. Using a hybrid method based on neural networks and Bayesian classifiers, their sensitivity to wounds and NT reached 78.7% and 86.3%, respectively. Zahia *et al.* 76 proposed a method using CNN for tissue analysis of pressure ulcer images. They extracted ROI from the raw wound images, removed noise, and extracted 5X5 pixel patches from each ROI for precise classification. This method achieved a classification accuracy of 92.01% for different tissue types such as GT, slough, and NT. In further research, Nejati *et al.* 77 divided chronic wound images into nXn patches and fed them into a deep neural networks (DNN) to extract features and train an SVM classifier. This approach achieved an accuracy of 86.4% in classifying seven tissue types (NT, slough, healthy GT, unhealthy GT, hypergranulation, infected, and epithelialized). In contrast, recent studies have proposed an automated wound assessment method by combining automatic color correction with DL models, such as variants of EfficientNet and MobileNetV2 integrated with U-Net architecture. This method effectively compares images under different lighting, distance, and camera conditions, excelling in segmenting wound areas and GT but still requiring improvements in ET and NT segmentation 78.

AI techniques, utilizing a multi-view strategy and superpixel FCN methods, have significantly enhanced the efficiency of wound tissue classification 79. Currently, image enhancement strategies have been used to improve the accuracy of tissue classification in chronic wounds^80^. Niri *et al.* 81 introduced a superpixel segmentation technique using linear iterative clustering (LIC combined with five-dimensional color (R, G, B, X, Y) and image plane space. By utilizing LIC with its powerful pixel segmentation and tight boundary fitting capabilities, fast and effective image segmentation can be achieved. The researchers inputted the above results into FCN Net based on VGG16 (including FCN-32, FCN-16, and FCN-8) and successfully segmented healthy skin and chronic wound areas, improving the accuracy of tissue classification and DC to 92.68% and 75.74%, respectively. Goyal *et al.* 82 employed superpixel color descriptions to obtain ROIs and inputted them into an integrated CNN model based on InceptionV3, ResNet50, and InceptionResNetV, achieving classification accuracies of 90% and 73% for ischemic and IT, respectively. Additionally, researchers have developed a computer-aided tissue classification scheme for chronic wound assessment using image processing and DL 83. In this scheme, wound images are converted from the original RGB to HIS, and a fuzzy divergence thresholding method is used for region segmentation to reduce edge interference. The study found that using an SVM with a cubic polynomial kernel could accurately classify GT, NT, and slough based on color and texture features, achieving an accuracy of 86.3%.

Using an app for remote wound tissue classification can help patients monitor wound dynamics in real-time, alleviating anxiety during home treatment. In telemedicine, researchers have developed a mobile wound capture system that uses smart device cameras to collect wound images. A new image database called “Complex Wound DB” has been designed to classify complex wounds into five categories: non-wound area, GT, fibrinous tissue, dry necrosis, and hematoma. Although consisting of only 27 images annotated by four health professionals, this dataset is publicly available 84. Currently, Shenoy *et al.* 85 have developed a mobile application named Deep-wound based on a multi-label CNN ensemble that can classify wound images, facilitating daily wound care. Moreover, remote automated wound tissue classification methods will provide valuable advice to doctors, aiding in more comprehensive wound assessment and more precise treatment planning. Table 4 provides a summary of wound tissue type classification.

Notably, a study using a U-Net architecture and the DL toolbox in MATLAB 2019a classified HE-stained images of mouse wound sections into GT, the epidermis, dermis, muscle, and background with accuracies exceeding 90%. However, classification accuracies for scabs and hair follicles were slightly lower 86.

Currently, wound tissue classification relies on analyzing features of each pixel or groups of pixels (superpixels) in the image and assigning them to different tissue types (e.g., GT, slough, necrosis). This method can address critical diagnostic issues. However, the high similarity between wound tissues, particularly between GT and infected tissues (IT), necessitates optimizing multi-view strategies and superpixel FCN methods. Building on this, combining surface wound images with pathological sections can further enhance identification accuracy.

## AI monitoring and prediction of wound healing

Predicting wound healing trajectories is particularly challenging but is crucial for achieving wound resolution 87. Predicting wound healing helps physicians select the most appropriate treatment plans, and enhances the efficiency and effectiveness of wound care. By automatically learning from and monitoring large volumes of clinical records, AI enables precise wound measurements from specific data sources and uses diagnostic data to predict wound healing trajectories 88-90. Furthermore, predicting wound healing times helps healthcare providers plan treatment strategies, set realistic patient expectations, and potentially improve outcomes while reducing costs.

### AI monitoring of cell behavior

**Figure 2:**
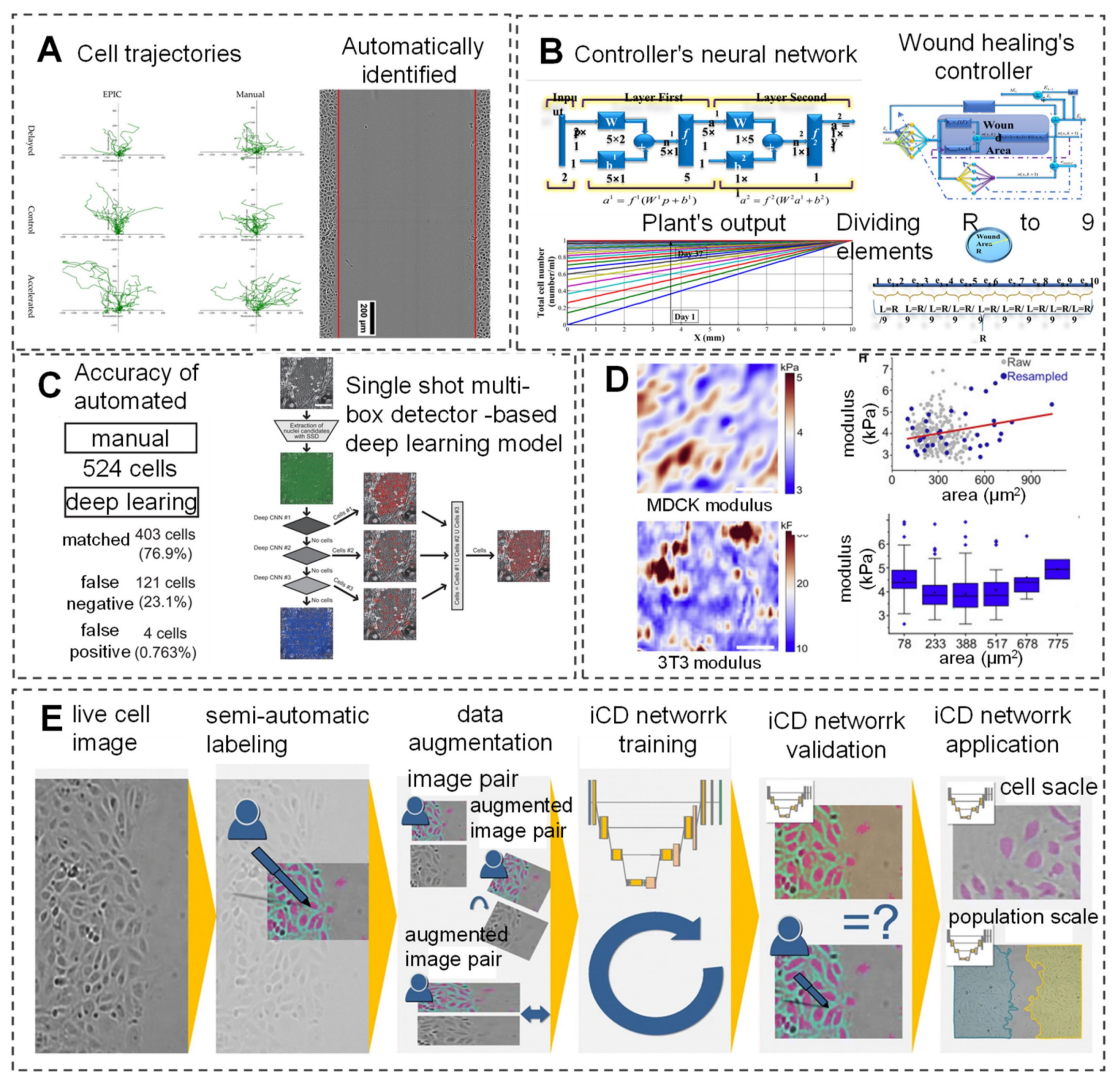
** AI is employed to observe cell behavior in wounds. (A)** Cell trajectories at the leading edge are monitored using manual cell tracking, EPIC, and Viterbi during experiments conducted at different speeds. Reproduced from ref. 92, an open access article 2022 published by MDPI under a CC-BY4.0 license. **(B)** An AI Controller, free from model limitations, is developed using output error to regulate wound healing Reproduced from ref. 93, an open access article 2017 published by Biosensors Journal under a CC-BY4.0 license. **(C)** Development of DL-based object tracking model for automated cell tracking in human keratinocyte colonies. Reproduced with permission from ref. 94. Copyright 2021 Oxford University Press. **(D)** AI can predict the effective modulus of MDCK and 3T3 cells. Reproduced with permission from ref. 96. Copyright 2022 Elsevier. **(E)** The process flow of a DL MATLAB application is illustrated to demonstrate the proper utilization of the ICD network. Reproduced from ref. 95, an open access article 2021 published by BioMed Central under a CC-BY4.0 license.

Wound healing arises from the coordinated actions of fibroblasts and epidermal cells, with cellular behavior mirroring the state of tissue repair. Simulating these behaviors enables monitoring of wound healing progress. A multi-agent heuristic technique, simulated fibroblast optimization, has been proposed to simulate fibroblast migration and systemic behavior, encompassing migration to connective tissue, collagen synthesis in the extracellular matrix, and new tissue formation during healing 91. While manual cell tracking techniques effectively determine cell trajectories and wound closure rates, they are time-consuming and prone to bias, limiting their utility in high-throughput experiments. To address this, EPIC software has been developed, employing AI to automatically track low-resolution, low-frame-rate cells. This software analyzes high-throughput drug screening experiments, yielding various wound healing metrics and publishable data (Figure 2A) 92. Additionally, researchers have devised a model-free controller for the wound healing process using a neural network controller (NNC). By training the NNC with an appropriate reference model and measuring skin cell content as the output, effective control of the healing process is achievable (Figure 2B) 93.

Recently, a new automatic cell tracking system named Deep-ACT has been developed, which combines cascaded cell detection with a Kalman filter-based algorithm to quantitatively evaluate single-cell movement and dynamics. Additionally, this system can distinguish the movement speed of cells in the central region compared to those in the peripheral areas (Figure 2C) 94. Oldenburg *et al.* 95 have developed an intelligent cell detection (ICD) method based on CNN, which demonstrates high robustness against image distortion. This method analyzes high-throughput drug screening experiments, generating various wound healing metrics and publishable data. It matches the speed of manual methods in detecting endothelial cell migration and is twice as fast in cell image speed measurement (Figure 2E). The mechanical modulus of cells significantly impacts cellular functions. Bermudez *et al.* 96 were the first to quantify the non-uniform deformation of mildly stretched cell layers and use AI inference to convert measured strain fields into effective modulus fields, allowing researchers to visualize the effective modulus distribution of thousands of cells (Figure 2D).

AI provides precise data support and decision-making for clinical practice in wound healing. AI can not only monitor and simulate cellular behaviors but also regulate the entire healing process through intelligent analysis and control techniques. Cell behavior detection heavily relies on image quality and user input. With advancements in technology and algorithms, it is anticipated that cell labeling and detection will become feasible, further enabling the *in vivo* monitoring and control of skin cell behaviors to predict and oversee the entire wound healing process.

### AI monitoring and prediction of wound progress

**Figure 3:**
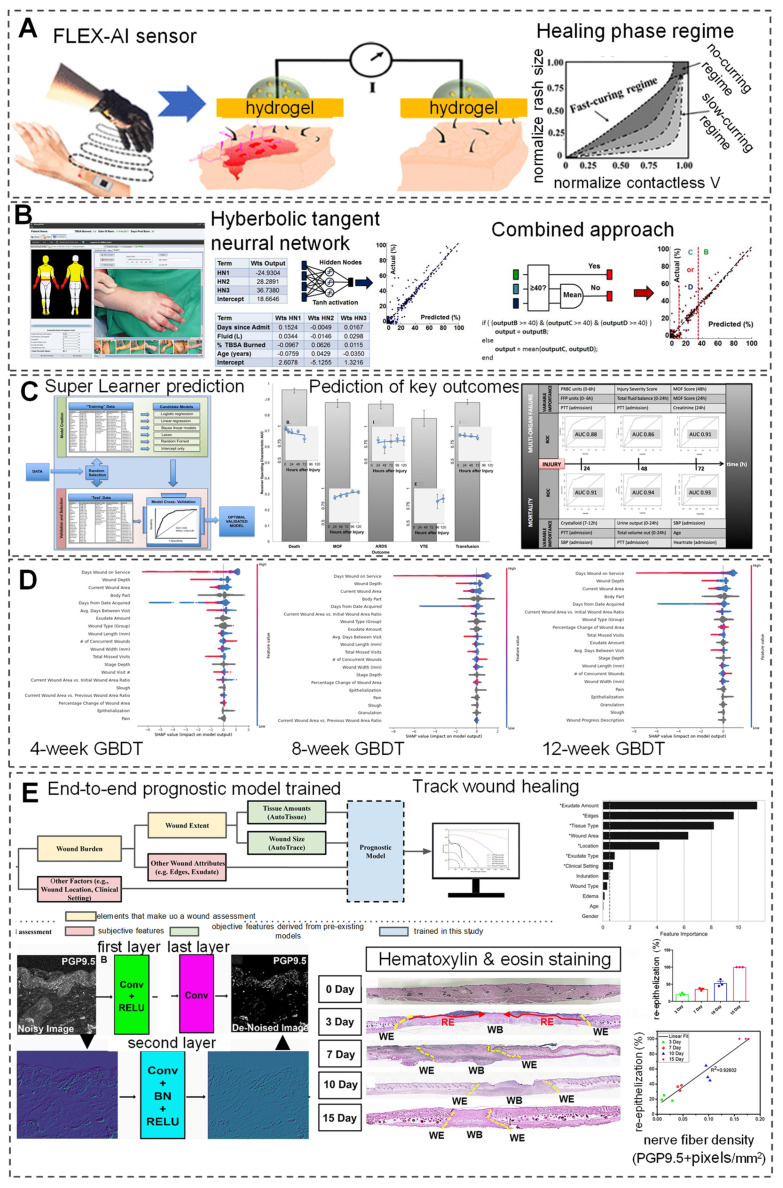
** The prediction of wound development through AI is explored in various studies. (A)** Advanced wound dressing bandages with intelligent wearable sensors for remote monitoring of chronic skin conditions Reproduced with permission from ref. 98. Copyright 2022 American Chemical Society. **(B)** Predicting wound healing capacity under any given burn size and fluid volume condition. Reproduced with permission from ref. 101. Copyright 2018 Oxford University Press.** (C)** Analysis of severe trauma dynamics using DL algorithms. Reproduced from ref. 102, an open access article published 2019 by Public Library of Science under a CC-BY4.0 license. **(D)** Prediction of chronic wound healing duration through DL with identification of the top 20 influential variables in the GBDT predictive model at 4, 8, and 12 weeks. Reproduced from ref. 106, an open access article published 2022 by Mary Ann Liebert under a CC-BY4.0 license. **(E)** Assessment of innervation in wound healing facilitated by DL. Reproduced from ref. 108, an open access article published 2023 by Springer Nature under a CC-BY4.0 license.

AI has shown immense potential in simulating and controlling the wound healing process, particularly during the remodeling phase. By simulating the healing process after burns, AI can provide scientific evidence for the true healing potential of each burn-imaging area 93. Furthermore, by training on large clinical datasets and real-time detection data, this technology can continuously and comprehensively monitor the pathological and physiological development of trauma patients, offering significant advantages over traditional triage tools and warning score systems, thus opening new avenues for trauma care. For instance, a study utilized an APP called Foot Snap on an iPad to observe plantar images and predict the incidence of DFU. This program standardizes foot images, evaluates the healing process of DFU (shape, texture, and color) through machine vision algorithms, and enables wireless real-time non-contact wound detection 97. Additionally, Kalasin *et al.* 98 developed AI-guided wearable sensing technology (FLEX-AI) by combining flexible wearable systems and deep artificial neural networks (DANN). This system using wireless communication technology combined with bandages containing dressings, trained with pH-responsive voltage output, achieved an accuracy of 94.5% in monitoring and treating chronic wounds (Figure 3A).

Traditional wound healing monitoring often does not consider treatment factors, making wound prognosis challenging. In a study, researchers evaluated the effectiveness of the Swift Skin and Wound technology in assessing wound healing under nutritional intervention. In the evaluation of 11 types of wounds, it was found that the system can effectively evaluate wound area with an ICC of up to 0.99, indicating excellent reliability in real-time tracking of healing progress 99. Regarding prognostic factors, Robnik-Sikonja *et al.* 100 employed the attribute estimation algorithms Relief F and RRelief F to evaluate and rank the factors that affect the wound healing process. The initial wound area, patient age, time from wound appearance to treatment initiation, wound shape, location, and treatment modality are important prognostic factors in sequence. Meanwhile, At the same time, researchers used an improved CORE learning system to construct a regression tree and combined it with prognostic factors to accurately predict wound healing rates, with an accuracy of up to 80% and 90% at 5 and 6 weeks, respectively. In another study, Liu *et al.* 101 evaluated the effectiveness of least squares regression models and ML in predicting open wound size in 121 patients and identified independent predictive factors affecting open wound area through goodness of fit statistical methods. Results indicated that ML can accurately predict wound size based solely on four factors: fluid volume, length of hospital stay, burn area, and age, with an absolute error of less than 4% (Figure 3B). Christie *et al.* 102 evaluated the ability of the integrated ML algorithm SuperLearner to dynamically assess severe trauma. In the analysis of 1494 severely injured patients, it was found that the algorithm can dynamically predict patients with severe trauma, avoiding a one-size-fits-all approach to trauma repair. Under ten-fold cross-validation, the algorithm achieved prediction accuracies of 0.94-0.97, 0.84-0.90, 0.87-0.90, 0.84-0.89, and 0.73-0.83 for posttraumatic death, multiple organ failure, blood transfusion, acute respiratory distress syndrome, and venous thromboembolism, respectively. This algorithm can help clinical doctors integrate large amounts of data from severely traumatized patients to make real-time, dynamic treatment decisions and predict patient outcomes (Figure 3C).

In clinical practice, tissue color changes are often utilized to assess wound healing status. Wang *et al.* 103 developed a smartphone wound image analysis system based on tissue color changes. The system uses an accelerated mean shift algorithm for wound segmentation and boundary detection to evaluate the healing process of DFU. Analysis of the images collected from the clinic revealed that the system is capable of high-precision and rapid evaluation of wounds, with a Matthews correlation coefficient of 0.736. In addition, researchers have stated that the system can run on both smartphones and servers simultaneously, which is crucial for remote healthcare and will allow doctors to diagnose and evaluate wounds through remote access. Additionally, to enhance accuracy, researchers have employed mask overlay to construct complete tissue layers, which realistically display different stages of wound repair. ChitoTech company has developed a mobile application called "Heal App" that utilizes AI to assess wound size, topology, shape, and color, and track wound changes. This application provides continuous monitoring in wound care centers and patients' homes, enabling clinicians to remotely track and obtain accurate wound information 104. Wang *et al.* 105 developed an integrated system that combines ConvNet and SVM to simultaneously perform wound segmentation and analysis, achieving fast wound segmentation and infection detection with a time and accuracy of 5 seconds and 95%, respectively. Moreover, when predicting wound healing, it was found that all wound outcomes predicted by the developed system were consistent with the average healing outcomes, at 95.67 weeks. Matthew *et al.* 106 developed a ML model based on gradient-based decision trees using EHR data, with an accuracy of AUC 0.854, 0.855, and 0.853 for assessing the risk of wound nonhealing at 4, 8, and 12 weeks. In addition, to further determine the factors affecting wound nonhealing, researchers used Shapley Additive Explanations to evaluate the output results of each factor and found that treatment duration, wound depth and location, and wound area were indeed the most influential factors (Figure 3D). Gupta *et al.* 107 trained a prognosis model, AutoTrace, using DL based on a dataset comprising 2,151,185 wound assessments and images. The model extracts objective features and subjective features such as tissue type, exudate amount, and wound area from images captured during wound assessment to predict wound healing quantification and progression patterns. Understanding these factors allows healthcare personnel to adjust wound care plans in real-time.

Additionally, Mehta *et al.* 108 employed a denoising convolutional neural network (DnCNN) to preprocess immunohistochemical (IHC) images and utilized automated image analysis to determine the positive correlation between nerve fiber density and re-epithelialization. This approach accurately assesses the degree of skin nerve innervation during various stages of wound healing (Figure 3E). Furthermore, DL algorithms are utilized to predict the risk of lower limb amputation in DFU patients, which provides clinical insights through interpretability, thus aiding early intervention to reduce the occurrence of reoperation and delayed healing rates 109, 110. The outputs of AI models involve objective measurements and predictions related to wound healing, which include quantifying healing rates, estimating healing times, or predicting the likelihood of successful healing. AI can identify key wound characteristics and patient-specific attributes such as age, as well as treatment plans, which suggests the potential for predicting post-treatment wound healing rates based on factors and information collected through surveys or electronic medical records 111, 112. In summary, the application of AI in wound healing demonstrates significant innovation and potential, not only in accurately monitoring and simulating cell behavior but also in regulating the healing process through intelligent analysis and control techniques. Through AI-enabled quantitative assessment of wound healing, healthcare professionals can track wound progression in real time, make informed decisions, and tailor treatment strategies based on predicted outcomes.

## AI-assisted personalized wound care and treatment

The application of AI impacts diagnosis, treatment, and prognosis, as well as workflow efficiency and expanding opportunities to access high-quality care. By enhancing the level of care provided by healthcare teams, the integration of AI can yield better outcomes for patients 113, 114. In a non-randomized controlled trial conducted on patients with lower limb ulcers, DFU, and pressure sores, AI medical devices could remotely collect precise wound data and automatically obtain objective clinical parameters with an accuracy rate of 97% 18. This capability of AI can be replicated across platforms for monitoring skin injuries, serving as an adjunctive tool to aid experts in assessing the condition of wounds. Although such systems may have errors, advancements in tools can correct these errors 115. The application of AI in wound care offers unprecedented possibilities for personalized treatment, facilitating optimized treatment plans and improved patient outcomes through precise data analysis and prediction.

### AI-assisted personalized wound treatment programs

**Figure 4:**
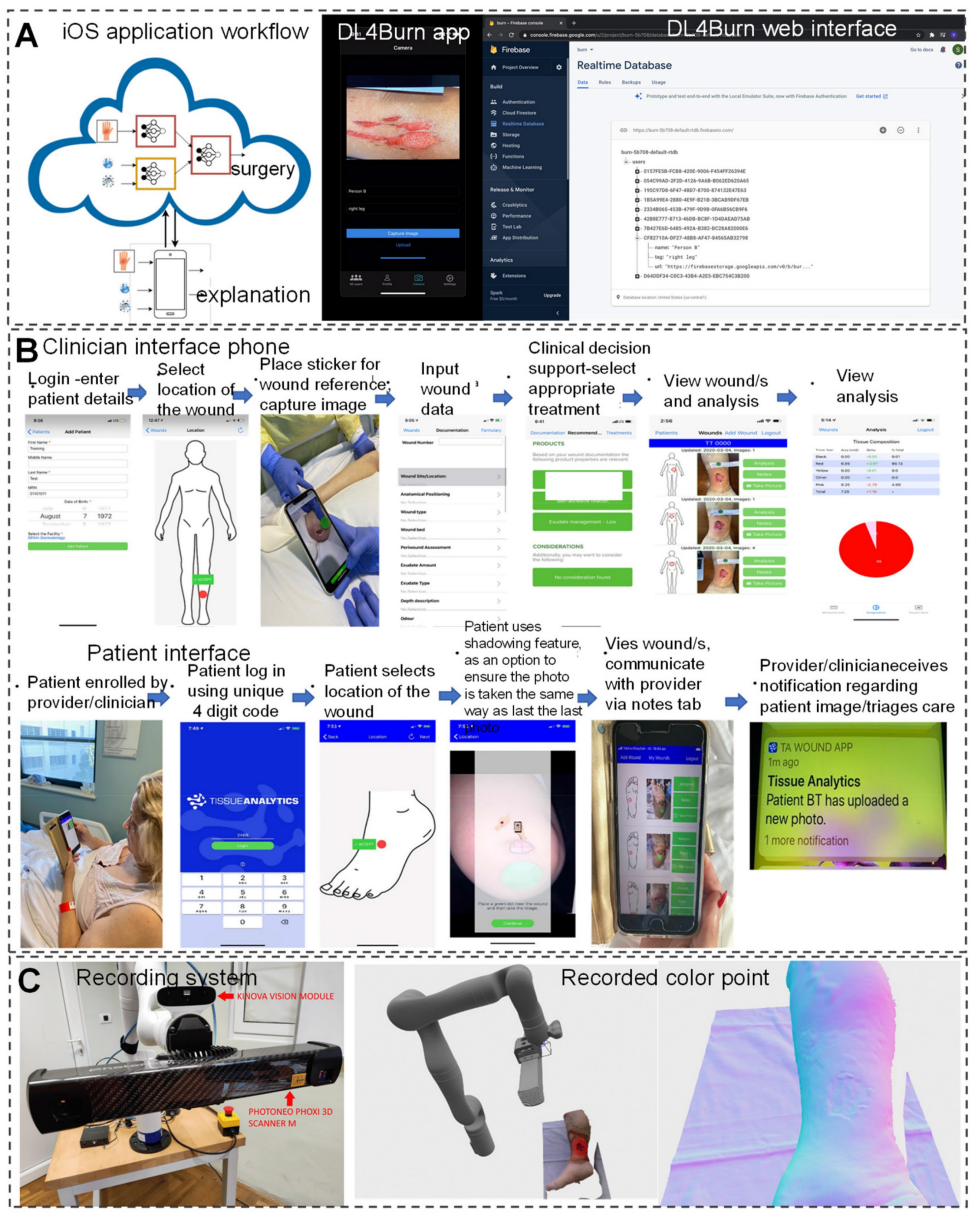
** Personalized wound treatment programs assisted by AI. (A)** DL4Burn utilizes DL to integrate various modalities for predicting candidacy for burn surgery, with details available on the iOS application and implementation via Google Firebase. Reproduced from ref. 118, an open access article 2022 published by American medical informatics association under a CC-BY4.0 license. **(B)** An AI application is developed to improve wound evaluation and treatment, featuring apps for both clinician interface and patient interface. Reproduced from ref. 119, an open access article 2022 published by Wiley under a CC-BY4.0 license. **(C)** An automated robot-driven system is introduced for reconstructing 3D images of chronic wounds. Reproduced from ref. 122, an open access article 2021 published by MDPI under a CC-BY4.0 license.

In the field of modern wound care, a comprehensive wound recognition strategy is crucial. By extracting specific geometric information from wounds to achieve precise identification, this strategy combines interdisciplinary technologies such as image recognition, computer modeling, and nanomaterials, enabling personalized and diversified clinical applications. For example, the integration of intelligent recognition and computer modeling technologies, along with the customization of materials, addresses the issue of excessive coverage by traditional dressings. This allows dressings to accurately cover wounds, reducing stimulation to surrounding normal tissues and accelerating the healing process 116, 117. The application of DL models in assisting burn wound surgical decision-making demonstrates further advancements of AI in the medical field. Researchers have developed the DL4 Burn mobile application using multimodal DL methods to simulate the multifactorial decision-making process of clinical doctors and predict the feasibility of burn surgery (Figure 4A) 118. Additionally, significant progress has been made in real-time data acquisition for wound monitoring and care using AI. Researchers evaluated the effectiveness of a ML-based Tissue Analytics application in promoting wound recovery. It accurately and objectively records and evaluates wounds, and builds a communication bridge between patients and healthcare professionals, which is crucial for wound management and recovery (Figure 4B) 119. Compared with the control group of patients, patients who received this program intervention showed significant improvement in wound recovery, with an average reduction of 53.99% in wound size. This indicates that AI technology has a significant promoting effect on the wound recovery process. AI chatbot software, as an auxiliary tool, can provide personalized treatment and lifestyle advice. By simply describing the conversation, the software can provide accurate wound care plans for 80 patients, with an accuracy rate of up to 90% (91% of patients). This indicates that AI technology has great potential in wound care, especially in complex wound management, in the future, reducing the time patients spend visiting hospitals while maintaining optimal wound care 120.

Furthermore, AI medical devices automate the acquisition of objective clinical parameters, enabling precise classification and tissue segmentation analysis of wound bed preparation (WBP). AI medical devices achieve an accuracy rate of 97% in WBP classification and tissue segmentation analysis, significantly improving the utilization of medical resources and the scientificity of treatment decisions 18. Novel portable handheld probes integrate 3D scanning, temperature measurement, multispectral, and chemical sensors for real-time wound diagnosis. They enable analysis of chronic wound tissue composition, area, volume, and temperature profiles, allowing for more accurate detection of wound environment changes and diagnosis of healing progress 121. Filke *et al.* 122 developed an intelligent robot system (HIS), comprising a free robotic arm, high-definition camera, and high-precision 3D scanner, achieving precise measurements and automatic recording using surface point density estimation and discontinuity detection. In hospitals, wound care nurses utilize the HIS system to obtain patient basic information, input wound diagnosis results and treatment processes, integrate wound data, and realize personalized management (Figure 4C).

The application of AI in wound recognition, monitoring, and care not only improves clinical efficiency and accuracy but also promotes the development of personalized medicine. In the future, with the widespread application of multimodal data input and advanced algorithms, AI-based wound diagnosis and treatment systems will be able to classify and analyze wounds more accurately, provide targeted treatment plans, and achieve personalized and precise wound care. To further standardize and optimize wound diagnosis and treatment, establishing a national minimum dataset and developing corresponding communication tools, such as the "Wound Care Log APP," are necessary steps to drive the development of this field. These measures will assist medical professionals in establishing unified standards nationwide, achieving interoperability of wound data, and providing patients with higher quality and more systematic wound care services 123, 124. However, AI-based applications face some challenges in clinical use and result interpretation, such as data privacy, improper or outdated data selection, selection bias, and historical biases, which may lead to erroneous conclusions. With an understanding of AI and continuous improvement, clinical doctors will be able to adopt this method more effectively to prevent and manage chronic wounds.

### AI-assisted develop personalized wound treatment products

In the field of modern medical technology, the application of AI is driving innovation in skin model construction, wound healing process simulation, drug development, and smart dressings providing new perspectives and tools for wound treatment.

**Figure 5:**
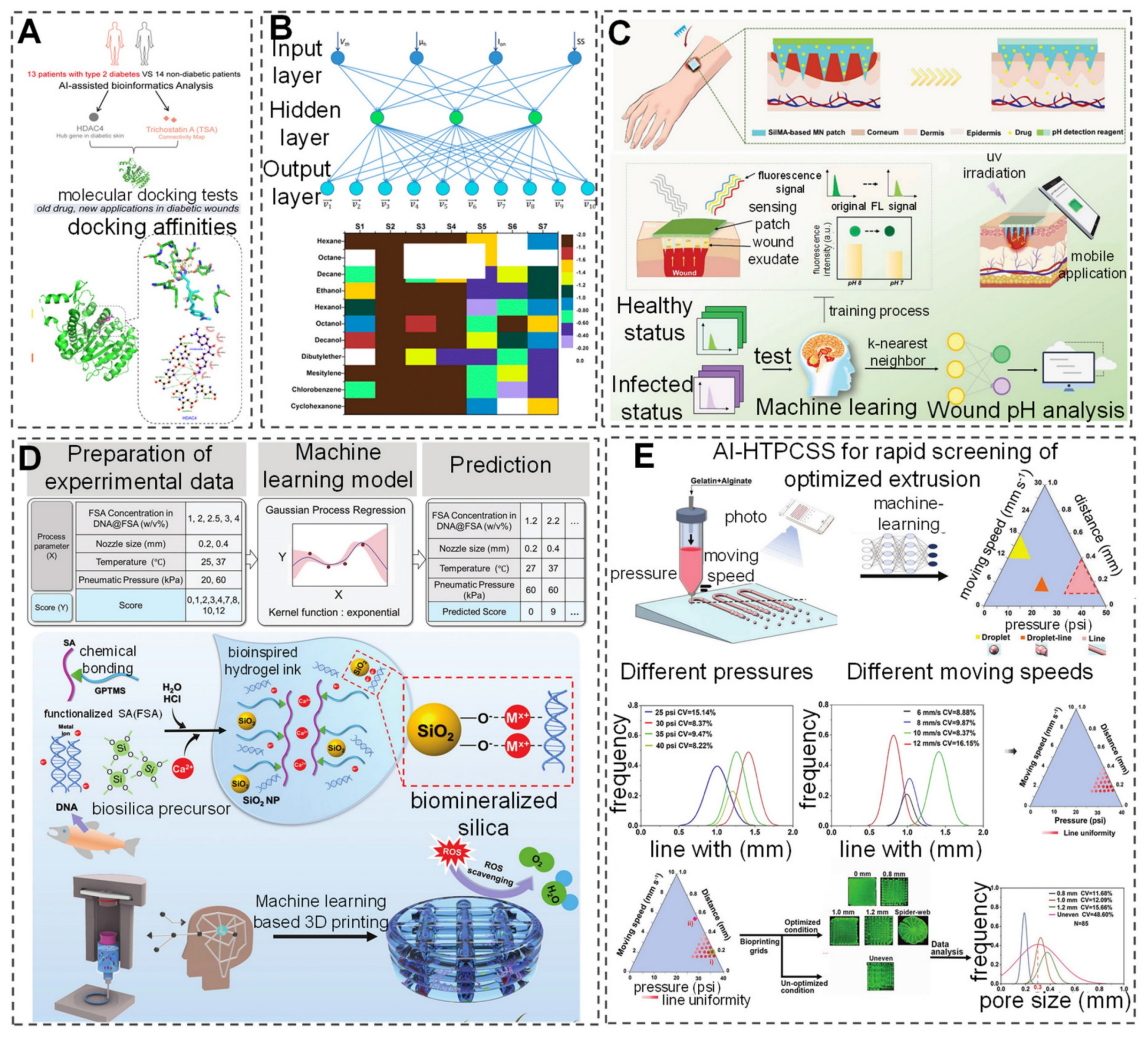
** AI-assisted develop personalized wound treatment products. (A)** AI-assisted bioinformatics analysis is employed to investigate the therapeutic potential of TSA and the hub gene HDAC4 in diabetic wounds. Reproduced with permission from ref. 128. Copyright 2022 American Chemical Society. **(B)** Ultraselective detection in the gas phase is achieved with artificial sensory intelligence utilizing silicon nanowires. Reproduced with permission from ref. 129. Copyright 2014 American Chemical Society. **(C)** DL aids in creating a self-sterilizing microneedle sensing patch for monitoring wound pH visually. Reproduced with permission from ref. 131. Copyright 2024 Wiley. **(D)** A DL model based on gaussian process regression (GPR) forecasts the printable performance of bio-inks. Reproduced with permission from ref. 133. Copyright 2022 Wiley. **(E)** AI predictions aid in assessing the printability of bio-inks, facilitating the production of high-quality 3D-printed hydrogel dressings. Reproduced from ref. 134, an open access article 2023 published by Wiley under a CC-BY4.0 license.

Currently, research has developed hybrid models that combine volume, membrane, and one-dimensional models to construct three-dimensional geometric and mechanical models of skin/subcutaneous complexes, capturing complex internal structures through an automated process 125. AI-built artificial skin models offer a new perspective for wound repair research and facilitate the applications of products with wound repair or monitoring capabilities to these models, thereby greatly advancing basic research in the field and improving product development efficiency 126. In drug development, AI applications have also demonstrated remarkable achievements. The application of AI in screening for novel antimicrobial peptides further promotes innovation from surface antimicrobial to deep drug development. Although most screening studies are still conducted *in vitro* and *in vivo*, integrating computational and statistical frameworks with DL models provides new directions for drug development 127. Additionally, research based on sequencing results from diabetic patient skin and AI-assisted bioinformatics has identified a potential therapeutic drug, Trichostatin A (TSA), and a potential target, histone deacetylase 4 (HDAC4), for diabetic wound repair (Figure 5A) 128. Researchers have also developed an AI-nanomaterial sensing system for ultra-selective detection of volatile organic compounds (VOC). This system utilizes functionalized modified silicon nanowire field-effect transistors combined with different salt molecules. After integrating with an artificial neural network (ANN) model, the sensor can identify 11 VOCs efficiently even under physical/chemical interference, offering a promising approach for detecting VOCs in wounds (Figure 5B) 129. Although there are few reports on AI-assisted nanomaterials as antibiofilm agents, previous explorations suggest that combining different AI applications with biofilms and wounds can lead to the development of more compact wound management devices 130.

Currently, wearable sensors assisted by AI have been developed for wound detection and management. Kalasin *et al.* 98 proposed a flexible AI-guided (FLEX-AI) wearable sensor that utilizes a DANN algorithm for chronic wound monitoring and short-distance communication. It communicates with seamless, MXene-connected, radiofrequency-tuned, and wound dressing-integrated (SMART-WD) bandages. Additionally, a DL-assisted microneedle sensor patch has been developed and trained on a dataset of fluorescence intensity data using the KNN model to achieve multivariate classification of wound infection types. By combining smartphone-captured fluorescence images, pH value can be visualized, enabling accurate and reliable wound management (Figure 5C) 131. The application of AI in the field of bio 3D printing is evolving, particularly in auxiliary roles and data-driven manufacturing within 3D printing fabrication 132. The integration of 3D printing technology with AI continuously enhances the precision, versatility, and compatibility of various materials 133, 134. Researchers have optimized DL models based on Gaussian process regression (GPR) to successfully predict the printability scores of bio-inks (Figure 5D) 133. Additionally, a study proposed an AI-assisted high-throughput printing condition selection system (AI-HTPCSS) after optimizing DL models. This system comprises programmable pneumatic extrusion bio-printers and AI-assisted image analysis algorithms, capable of predicting the printability of bio-inks and subsequently optimizing the printing process to develop higher-quality three-dimensional printed hydrogel dressings (Figure 5E) 134. Through DL methods, the optimization of the 3D printing process of bio-inks has been achieved, providing new insights for developing superior quality three-dimensional printed hydrogel inks.

With the combination of advanced AI and the development of new materials, the field of wound treatment is experiencing unprecedented innovation and breakthroughs, providing vast development opportunities and potential for future medical advancements. With the further application and development of AI, it is expected to optimize wound diagnosis and treatment processes, combined with the development of personalized treatment products, to bring more precise and personalized medical services to patients, and improve treatment efficiency and effectiveness.

## Discussion and Perspectives

### Advantages and limitations of AI models and algorithms

Wound care involves tasks such as image analysis, tissue classification, size measurement, and temporal monitoring, which are often time-consuming and prone to assessor biases 21, 135. AI is revolutionizing traditional wound diagnosis and management methods, enabling greater precision and intelligence in the evaluation process 19, 136. A comprehensive AI-based wound diagnosis, prediction, and treatment system can significantly save clinicians' time, reduce patients' financial burdens, and improve their quality of life. Currently, AI-assisted wound diagnosis and treatment systems create an integrated framework by automatically identifying, analyzing, summarizing, understanding, learning, planning, and updating, thereby continuously assessing the entire wound healing process 19, 136. Despite the immense potential of AI in enhancing the safety, accessibility, and quality of wound care, the field is still in its early stages, and its clinical feasibility remains to be validated 21, 137.

In Table 5, we summarize the applications, advantages, limitations, and scalability of current AI models and algorithms in wound healing. Shallow-ML models, suitable for various data types, including structured and unstructured data, can identify and leverage the most relevant features to improve prediction accuracy. Algorithms such as random forests and SVM are used to predict healing outcomes by analyzing patient records and treatment data, and optimizing treatment plans based on specific patient characteristics (e.g., age, diabetic status, infection risk). However, shallow-ML models are prone to overfitting in small sample datasets, leading to poor performance on new data 65, 102, 103.

DL models, by learning from large datasets, achieve high classification and prediction accuracy, with CNN particularly excelling in wound image recognition and classification. These models can automatically identify wound types, assess healing stages, and estimate wound area. Additionally, DNN combine patient history data and wound characteristics to predict healing time. However, DL models require extensive labeled data for training, and their performance may be inadequate without it 46, 69, 81, 105, 138. Natural language processing (NLP) technologies, such as GPT, can efficiently process and analyze large volumes of text data, enhancing the retrieval of clinical information. By understanding and analyzing natural language, these technologies can extract key wound care-related information from EHRs for decision support 139, 140. By appropriately selecting and applying AI models, integrating image data with clinical data, and combining multiple models and algorithms, more efficient and precise diagnosis and treatment can be achieved in wound healing, ultimately improving patient outcomes and quality of life 106, 138.

AI applications in wound treatment are mostly in the research or small-scale clinical trial stages, and effective implementation in clinical practice requires further investigation 19, 21. We recommend the following: First, ensure compatibility between AI tools and existing Hospital Information Systems (HIS) and EHR systems for seamless data integration. Second, develop intuitive user interfaces and detailed training programs for AI technology to ensure that medical and technical staff acquire the necessary skills. Next, annotate data and use algorithms such as CNNs to train AI models.

For example, CNNs or SVMs can automatically identify and analyze wound types, sizes, and depths; NLP can parse physician notes and patient records to generate automated reports and recommendations 19, 52, 141. Validate model accuracy and practicality through cross-validation and clinical trials to ensure applicability in real-world settings. Finally, assess AI technology's effectiveness using specific clinical data, such as diagnostic accuracy, treatment success rates, and patient satisfaction, and refine technology based on feedback. Regularly update AI models to enhance performance and reliability. Furthermore, AI models must operate within legal and ethical frameworks, emphasizing interdisciplinary collaboration to ensure data security and compliance. In the future, AI technology will not only provide more precise support for wound care but also advance the development of personalized medical solutions, thereby improving patient health management and quality of life comprehensively.

### Challenges and coping strategies

AI requires extensive data support, but the lack of standardized wound data collection guidelines and protocols has led hospitals to develop wound data collection methods based on their specific needs, complicating the establishment of comprehensive wound care databases. Researchers face challenges in obtaining large and comprehensive wound care datasets, hindering the progress of AI in this field. Even with comprehensive datasets, unspecified or unmeasured factors may still influence wound healing outcomes, leading to incomplete or inaccurate predictions by DL models. Therefore, there is a need to establish standardized wound data collection systems and user-friendly recording devices to enhance operability and facilitate rapid wound care documentation across various settings. To expedite the wound diagnosis process, optimize treatment plans, and improve treatment efficiency and patient quality of life, we need to explore the integration of computational methods and AI to achieve these goals 135. However, the role of AI in automating wound diagnosis and healing prediction remains underexplored. It is crucial to identify suitable wound diagnosis tools and further standardize the testing and use of AI-based digital wound evaluation tools. This will help determine which wound types are best suited for AI management and which specific algorithms are most effective 142. Develop classification models tailored to specific wound types for detailed assessments, such as the severity of DFU or burn area. Foster inter-institutional collaboration to establish multicenter image databases, improving model generalizability. AI models can also collect patient health data, such as blood glucose levels and nutritional status, while regularly analyzing wound images to generate healing trend charts and predict wound healing times. These insights can assist clinicians in optimizing treatment plans, ultimately improving patient outcomes.

AI in wound identification is mainly limited to color recognition and lacks the predictive ability for wound flexibility and exudate information. Emerging devices cannot measure longitudinal wound information, as wound sinuses and fistulas cannot be scanned, requiring manual measurements to obtain data. This indicates the need for new strategies to collect, analyze, and process vast amounts of information to address these limitations. A recent study introduced the iDr smartphone application based on a mobile platform. The application utilizes low-cost yet accurate 3D imaging technology to comprehensively measure wound area and depth, with its relative error in wound area and depth measurement significantly lower than traditional clinical methods, offering new possibilities for remote healthcare and self-monitoring. This innovative solution eliminates the need for expensive industrial-grade 3D cameras and achieves non-invasive volume measurement economically and efficiently 143. Many studies have not yet evaluated the long-term effects of using AI applications, such as wound healing speed and quality. However, a few preliminary studies have shown high satisfaction with AI in clinical practice among both clinicians and patients 144. Future research should focus on deeper analysis of clinical outcomes, particularly incorporating patient feedback, while leveraging data augmentation and transfer learning to enhance model accuracy. Building on this, AI systems can be integrated with EHRs to combine multidimensional data, including patient history, lifestyle habits, and metabolic status, alongside wound-specific features such as temperature and color changes 135. This integration enables automated analysis of wound types and comorbidities. Based on historical data, AI can generate personalized care recommendations, such as wound cleaning frequency, dressing selection, and medication dosage, to support clinical decision-making and improve patient recovery management.

As experts say, "Treatment is not just the wound on the patient, but the whole patient." Thus, a comprehensive approach must consider a complex network of factors beyond just the wound 145. Integrating AI technology into existing clinical workflows and EHRs could address this complexity 111. However, this integration faces several challenges, particularly in managing trauma patients. For instance, current AI systems cannot automatically generate EHRs, and the extensive textual data they contain still requires manual entry by clinicians. Large language models (such as GPT) offer new possibilities for automated case generation. However, AI technology currently cannot personalize clinical pathways and treatment plans, which must still be developed by physicians. Google's Med-PaLM model offers new directions for managing personalized medical plans 146. Additionally, compatibility issues between AI tools and different EHR systems used by hospitals and clinics hinder data integration and consistency. AI systems require ongoing updates and maintenance to adapt to new medical knowledge, technological advancements, and regulatory changes, which demands long-term technical support and resources from healthcare institutions. In addition, the widespread application of AI technology in wound care faces several scalability challenges, including data quality and diversity, system integration, clinical workflow optimization, regulatory compliance, data protection, cost-effectiveness, and regional adaptability. To address these challenges, it is essential to adhere to local laws and regulations while establishing cross-institutional data-sharing platforms to collect high-quality, multi-source, and diverse data to enhance model generalization. Open standards and interfaces (such as HL7 and FHIR) should be adopted to promote system integration, ensuring that AI systems are compatible with diverse EHR systems and optimizing clinical workflows 147, 148. Regarding costs, cloud-based AI solutions should be promoted to reduce hardware investment and alleviate the financial burden on healthcare institutions. In resource-limited regions, low-cost and user-friendly AI tools should be used, and the technology's accessibility and application can be expanded through telemedicine and mobile health platforms.

As clinical diagnostics, prognosis, and treatment demands increase, integrating multimodal wound data (such as 2D images, 3D surface morphology, texture, text data from clinical records and EHRs, and even proteomics and genomics) for systematic wound management will become essential 19, 21. There is an urgent need to identify and guide methods for analyzing multimodal data to support continuous advancements in data processing capabilities. Additionally, data-driven technologies like DL could be utilized to build comprehensive public wound datasets with detailed annotations and develop AI-based evidence-based decision support systems to advance wound research. Future AI research should focus on identifying "wound patterns" by understanding the complex interactions between wounds and patient factors. Integrating multimodal datasets and mobile devices to develop remote intelligent wound care systems could improve clinical decision-making in wound diagnosis, treatment, prognosis, and management, ultimately significantly enhancing patient medical experience and quality of life.

### Ethical and legal challenges and strategies

AI diagnostics face challenges in technology, philosophy, law, and ethics, requiring active involvement in the development and application of AI technology. To fully leverage AI's potential, a balanced framework must be established to ensure responsible and fair use of AI in clinical practice. This includes strengthening the regulation of AI systems, addressing privacy protection, algorithm fairness, decision transparency, accountability, and regulatory compliance in wound care while maintaining control in the evolution of human-AI collaboration 149. First, ethical review must be strengthened. In AI applications for wound care, patients should have the right to understand and decide whether to consent to AI involvement in their treatment process. Ensuring informed consent not only meets ethical standards but also enhances trust in AI systems. To achieve this, a standardized informed consent process must be established, providing a clear consent form before patients use AI-assisted systems, detailing the role, limitations, and potential risks of AI 150. Additionally, educational materials and explanations from healthcare providers should be used to communicate the application and limitations of AI in care, thereby increasing patient awareness and acceptance of AI-assisted treatments.

The application of AI technology involves vast amounts of patient data, raising significant concerns about data privacy and security. Therefore, not only must technology providers implement robust data protection measures, but healthcare institutions must also conduct compliance reviews and enforce protective measures. Robust AI technology must ensure the secure transmission and storage of sensitive health data, complying with strict privacy regulations such as the General Data Protection Regulation (GDPR) and the Health Insurance Portability and Accountability Act (HIPAA) 151. Obtaining explicit patient consent before data collection is necessary, alongside regular security audits and real-time monitoring of AI systems to ensure their safety. Moreover, secure AI systems should be designed, and staff should receive data protection training to effectively safeguard sensitive patient information and ensure the safe application of AI technologies 144. The development of AI systems requires significant initial investment, data collection, and long-term research, highlighting the importance of establishing digital research platforms and reasonable data collection guidelines for the success of AI research 152. Some companies collaborate with generative AI to provide research practice communities, building personalized information repositories through interaction and content sharing, thus offering researchers more specialized resources.

The quality and representativeness of training data are crucial in developing AI technologies, as biases or misjudgments can lead to unfair treatment in wound care, affecting treatment outcomes 153. To prevent this, training data should be diverse, covering various age groups, genders, and ethnicities, to minimize the risk of algorithmic bias 154. In practice, the algorithm's results should be regularly assessed, and biases corrected promptly to ensure fairness. During the design and validation phases, fairness testing should be incorporated, and fairness should be prioritized throughout the algorithm development process. Additionally, collaboration with ethics committees and multidisciplinary teams is essential to review datasets and algorithms, ensuring consistent accuracy across different patient populations.

In clinical practice, the transparency and interpretability of AI systems are considered fundamental to building trust. This requires clear communication to both physicians and patients about the limitations and confidence levels of AI diagnoses or predictions, and the development of usage protocols for wound care AI in collaboration with ethics experts. Moreover, AI systems are often "black box models," particularly DL models, where the decision-making process is difficult to explain. The lack of interpretability may affect patient consent to AI-assisted diagnosis and treatment. Therefore, AI systems should be interpretable, incorporating technologies such as LIME (Local Interpretable Model-Agnostic Explanations) or SHAP (Shapley Additive Explanations) to help physicians understand the rationale behind AI outputs. This allows healthcare providers to review the decision-making logic of the AI at any time, enhancing trust and acceptance of the system 155. Healthcare professionals must learn to interpret and use AI outputs to ensure they align with clinical standards and do not interfere with clinical judgment.

Given that AI predictions and recommendations may directly influence wound treatment plans, clear responsibility attribution is crucial. When AI causes misjudgments or biases that harm patients, responsibility must be clearly defined, with a "shared responsibility" mechanism in place to ensure that AI developers, healthcare institutions, and users assume legal responsibility within their respective roles 156. Operational standards and usage protocols for AI in wound care should be established, ensuring that healthcare providers receive training before using the system and understand their responsibilities regarding AI outputs. In clinical practice, a comprehensive accountability system should be implemented, documenting each step of the AI system's operation and the basis for its decisions, ensuring traceability of any issues. Meanwhile, efforts should be made to enhance public understanding and trust in AI technology through education and communication, increasing acceptance of AI's role in healthcare. Ultimately, the successful application of AI technology requires the collective efforts of governments, healthcare institutions, technology companies, and patients to ensure the harmonious development of technological innovation, ethics, and law 157.

### Socioeconomic impact and medical changes

The introduction of AI will reshape the roles of healthcare professionals. For example, tasks traditionally performed manually by doctors and nurses, such as wound assessment, monitoring, and treatment decision-making, will be partially taken over by AI automation systems. AI-driven wound assessment systems can provide real-time, accurate information on wound size, type, and healing progress, helping medical staff develop treatment plans more quickly. However, the widespread use of this technology may shift the role of healthcare professionals from direct intervention to supervision and decision support, requiring them to have the skills to interpret and manage AI recommendations to effectively use AI-assisted medical tools. As AI becomes more widespread, healthcare workers will face the need to learn new skills, particularly in data analysis, AI system operation, and data ethics 158. Furthermore, healthcare providers must enhance their understanding of AI's limitations and potential biases, and effectively explain AI's diagnostic recommendations to patients, alleviating concerns about "machine diagnosis" and minimizing the negative impact of AI systems in practice. While this retraining demand may pose a challenge to healthcare institutions' resource allocation, it also presents an opportunity to improve the overall skill level of healthcare professionals.

It is important to note that the widespread adoption of AI could reduce the workload of some positions in the healthcare field while increasing the demand for technical professionals such as data scientists and AI system administrators. The introduction of clinical decision support systems may reduce the need for human labor in repetitive or lower-complexity tasks, while advanced care and the management of complex cases will still require the judgment and expertise of healthcare professionals. Therefore, the application of AI not only impacts staffing and resource allocation but may also drive an increased demand for technical talent across the industry, promoting cross-disciplinary collaboration. Furthermore, the implementation of AI may require significant investment in infrastructure, particularly in AI devices, algorithms, and data storage. However, integrating AI offers opportunities to improve efficiency and the precision of patient care. For example, AI systems can reduce repetitive tasks for healthcare providers, allowing them to focus more on patient care that requires interpersonal interaction, thereby improving both medical efficiency and patient experience 159. Nevertheless, the core of healthcare remains patient-centered care. In some cases, patients prefer direct interaction with healthcare providers over relying on machines for decision-making. Therefore, while promoting AI technology, it is crucial to ensure that healthcare professionals remain involved in the diagnostic and treatment process to maintain patient trust and satisfaction 160. Ultimately, a balance must be struck between improving efficiency and ensuring care quality, ensuring that AI applications do not undermine the human interaction in healthcare.

With the advancement of AI and the accumulation of data, AI applications not only provide high-quality home care for patients but also drive the development of remote medical systems, offering professional technical guidance to patients in remote areas. AI systems can remotely measure wounds and communicate with online commercial organizations regarding healing trajectories, integrating them into remote medical platforms to provide care in resource-limited environments 119. The increase in massive data and powerful computational resources has played a crucial role in data-driven care through AI-based digital platforms, reducing expert workload, increasing accessibility to professional knowledge, and expanding the potential for remote wound treatment. Especially during the COVID-19 pandemic, computer-assisted remote wound care technologies have accelerated 161. Utilizing tools like ChatGPT, complex tasks can be completed within seconds, enhancing efficiency and enabling researchers to focus on actual research rather than tedious administrative tasks. Through generative AI collaboration platforms like ChatGPT and AlphaFold3, researchers can exchange and share research content, transforming personalized content into individual data or research repositories, and providing more specialized outputs for generative AI. These specialized databases are more accurate than traditional internet methods, saving researchers time and providing outputs for educating others 162.

In conclusion, while developing industry consensus or guidelines is still a long way off, professional clinical guidelines will help standardize and improve the application of AI in wound care, addressing issues of inconsistency and accessibility. Stakeholders, including technology providers, healthcare institutions, and policymakers, must collaborate to create an environment conducive to AI development, promote more efficient and safe medical services, enhance patient care quality, optimize medical decision-making, and improve operational efficiency. Looking forward, collaboration among multidisciplinary teams-including clinicians, scientists, and regulatory experts-is crucial for bridging the gap between research outcomes and the clinical application of burn and wound healing. As AI systems continue to optimize, they will adapt to cases involving different regions, races, ages, and trauma characteristics, providing scientific support for wound diagnosis and treatment, reducing mortality and complication rates, improving treatment quality and efficiency, and alleviating social burdens. Although AI has demonstrated significant potential in healthcare and is beginning to challenge the professional role of doctors, it cannot replace the diagnostic capabilities of physicians 163. Clinicians should enhance their medical skills and avoid over-relying on AI. Ultimately, it is essential to recognize that no tool can fully replace direct clinical diagnosis. This underscores the importance of a human-AI collaboration model, where AI is not viewed as a replacement for human labor but rather as a tool to enhance healthcare services 164, 165.

## Conclusions

This article reviews the research status of AI applications in injury classification, wound measurement (area and depth), wound tissue classification, wound monitoring and prediction, and personalized treatment. It summarizes the datasets used, development methods, and algorithms, and lists the limitations and future directions of AI, providing scientific evidence and technical support for further development of AI in wound treatment and care. In conclusion, there are many opportunities for the application of AI in wound theranostics, including standardization of treatment, patient self-management, optimization of healthcare workflows, personalized treatment plans, and improvement of education and awareness between patients and providers. Future collaboration between wound care professionals and AI researchers is needed to advance translational medicine, improve human health, and reduce healthcare costs.

## Figures and Tables

**Table 1:** Wound type classification

Classification	Used Method(s)	Used Features	Dataset	Outcome	Advantage or Limitation	Ref.
DFU	DL algorithm (DFUNet), aspect of CNNs architecture-depth and parallel convolution layer	Differences of DFU and healthy skin patches	Extensive dataset of foot images	AUC score: 0.961	Cost-effective, remote, and convenient	23
DFU	A cascaded two-stage approach based on ML implemented with a SVM	Color and texture descriptors from superpixels	100 DFU images	Sensitivity: 73.3%, Specificity: 94.6%	Need to be expanded in wound image database	24
DFU and VLU	DCNN with pre-trained weights	Standard augmentations of images and pixels; Wound characteristics and pixels	863 images from wound care centress	FI:0.85	Need to be expanded in terms of wound images and wound types; Few wound images and wound types	25
Various injury occur in a disaster site	DL algorithm (RTS, logistic, random forest, DNN)	460,865 cases: vital signs and a consciousness index	National trauma databank	AUC scores: (RTS: 0.78; Logistic regression: 0.87; Random forest: 0.87; DNN: 0.89.)	Reduce triage time; Retrospective study; Deficiency of samples collected	26
DFU, Lymphovascular, Pressure, Surgical	Explainable AI (transfer learning, data augmentation and DL)	Wound images are then hand-labeled	8690 wound images (1811 DFU, 2934 lymphovascular, 2299 pressure injury, 1646 surgical wound images)	FI: 0.76	Provides chronic wound classification and its associated explanation	27
NU, surgical site infections, VLU, pressure	DenseNet, MobileNet and ResNet	A large number of wound images from a multi-ethnic population	2957 from image registry tertiary institution Singapore	Accuracy: 96.3%; F1: 0.96	Development of an explainable AI model for Asian	28

Artificial intelligence: AI; machine learning: ML, deep learning: DL; area under the curve: AUC; support vector machine: SVM; deep neural network: DNN; deep convolutional neural networks: DCNN; diabetic foot ulcers: DFU; pressure ulcers: PU; venous leg ulcers: VLU.

**Table 2:** Wound area measurement

Type	Used Method(s)	Used Features	Database source	Outcome	Limitation	Ref.
Ulcer, plastic wounds	OPEN CV	Grayscale and DPI	Ten images captured using different kinds of devices	Average MEM of 4.4% for high quality pictures	Greatly affected by shooting distance, brightness and noise	31
All types of wounds	Swift Skin and Wound mobile application	Wound margin and length and width	177 wounds in 56 patients	_-	Difficult to generalize and lack high sensitivity	39
PU	Burn Case 3D program	Interest wound region provided by the structure sensor and length and width	232 pressure injury photos	_-	Affected by the photo taking and the operator	40, 41
Sprague-Dawley rats skin defect mode	Interactive Graph Cuts algorithm	Interest region of depth-map provided by the structure sensor	A total 46 wounds, including 32 irregular wounds and regular 14 wounds	All correlation coefficient exceeds 0.93	High reference value for monitoring the process of wound healing; Animal skin defect mode	42
Burn	ResNet101, U-Net and Mask R-CNN	Standard augmentations of images and pixels	3571 images of burns from Far East Hospital	DC of ResNet101: 0.9496	Fewer training images	43
PU, DFU and VLU	Seg Net, LinkNet, U-Net, U-Net-VGG16	-	400 PU, 20 DFU,and 20 VLU	VGG16 with highest accuracy	Race bound	44
DFU	A CNN-based method for extraction of diabetic lesion regions	Pixels from noise-removing images	341 images	Pixel accuracy: 0.934	Fewer training images	45
DFU and PU	An annotation tool based on watershed algorithm (FCN)	Standard augmentations of images and pixels	950 digital images	Accuracy: 98.12	Need to further expand the application	46
DFU	A composite wound segmentation framework (CNN)	Standard augmentations of images and pixels	950 wound images	Accuracy: 94.69	Difficulty distinguishing between non-skin backgrounds	47
DFU	A DFU image segmentation algorithm based CNN (Mask R-CNN)	Label of ulcer and image background	1176 images	Accuracy over 93%	Further validation using a framework	48
Burn	A segmentation framework based on the Mask R- CNN	Wound margin	1150 images from Genetic Engineering and Biotechnology	Accuracy: 84.51%	Fewer training images	49
Lower Limb Chronic Wound	An image segmentation algorithm based on a DCNN	Image features	112 cases	Total effective rate: 92.86%	Fewer training images and samples	50
DFU	Novel convolutional framework based on MobileNetV2(CNN) and connected component labelling	Pixels and shape from noise-removing images	1109 DFU images	DC: 90.47%	Extend to mobile devices in the future	51
Pressure injuries, arterial ulcers, and VU	DCN and DNN	Wound boundaries; Pixels and shape from noise-removing images	58 wound images consisted of 465,187 image-label pairs	Interrater agreement intraclass correlation 0.861 to GT	Poor ability to classify ET	52
DFU	Semantic segmentation of small datasets (SegNet and V-Net)	Pixels and color and thermal information	92 images	Dice score: 97.25%	Not consider 3D structures	53
Burn	Mobile application: BurnMed	Wound size	Burn on a mannequin	Measurement errors: -0.96 (3.74)	Mannequin not reality wound	56
VU	iPad app: WoundAide	Wound size	Six patients with 10 VU	Coefficient of variation: 3% to 33.3%; capturing sensitivity: 75%	Few patients; Least sensitivity in image captures	57
VU, DFU, surgical	Mobile application: Swift with HealX	Color, lighting and size	91 patients with 115 wounds	-	Limited clinical setting	59
Ulcer, lastic wounds	Mobile application : Swift Wound	Orientation and location of the wound margin	87 patients	ICC: 0.97-1.00	Differences between the training model and the actual verification	61
DFU	CARES4 WOUND system	Wound margin	341 wound images	Inter-rater reliability: 0.947	Accuracy depends on camera	62

Diabetic foot ulcers: DFU; arterial ulcers: AU; venous ulcers: VU; pressure ulcers: PU;venous leg ulcers: VLU; neural networks: NN, deep learning: DL; electronic health records: EHRs; area under the curve: AUC; support vector machine: SVM; convolutional neural networks: CNN; deep neural network: DNN; deep convolutional neural networks: DCNN; epithelial tissue: ET; granulation tissue: GT; intraclass correlation coefficient: ICC; Dice coefficient: DC.

**Table 3:** Wound depth measurement

Type	Used Method(s)	Used Features	Dataset	Outcome	Advantage or Limitation	Ref.
Burn	Build a classification model via DL (SVM and KNN classification); Z-test and univariate analysis to remove outliers	Pixel intensity and location of image	Multispectral imaging training database (single wavelength images)	Accuracy: 76%; Test accuracy was improved from 63% to 76%	A model based on swine; other descriptors of light-tissue interactions were not incorporated	65
Burn	A novel artificial burn depth recognition model based on CNN (ResNet50)	Standard augmentations of images and pixels and Patches	484 early wound images	Accuracy: 80%	Fewer training images	66
Burn	CNN-based algorithm and explainable AII (XAI)	Physical textural features in ultrasound and pixels in RGB images	10,085 frames of pigs and 338 images from web	Lobal accuracy greater than 84%	The influence of race or skin pigmentation on segmentation accuracy could not be assessed	67
Burn	DNN (VGG-16, GoogleNet, ResNet-50, ResNet-101)	Extracted interes regions and pixels	23 burn images	Average accuracy for the four different types of burn depth 90.54%	Fewer training cases	35
Burn	FCN	Coarse and pixels	180 images	Pixel accuracy: 0.60; IoU of 0.37	Class imbalance	69
DFU	Bilinear CNN (Bi-CNN)	Standard augmentations of images and pixels	1639 images	Accuracy: 84.6%	Class imbalance	70
Burn	End-to-end framework based on DL method	Standard augmentations of images and pixels	516 burn images	IoU: 0.5144; PA: 0.6684; DC: 0.6782	Fewer training cases	71
Praediatric scald injuries	CNN, based on the U-Net	Standard augmentations of images and pixels	100 burn images	Accuracy and DC, both on average 92%	Fewer training images and final healing result	68

Diabetic foot ulcers: DFU; intersection over union: IoU; Dice coefficient: DC; convolutional neural networks: CNN; support vector machine: SVM; k-nearest neighbors: KNN; fully convolutional network: FCN.

**Table 4:** Wound tissue type classification

Classification	Used Method(s)	Used Features	Dataset	Outcome	Limitation	Ref.
DFU	A generic Bi-CNN network architecture	Standard augmentations of images and pixels	A DFU dataset of 1639 images	Accuracy 84.6% for GT	Class imbalance	70
Surgical, DFU, and VU	DCNN-based classifier	ROI and patches	AZH Wound and Vascular Center: 400 wound images	Maximum accuracy: 96.4%; Average accuracy: 94.28%	Less financial and time costs	72
PU	NN, bayesian classification and SVM	Color and textural features	Color photos of PUtaken by clinical doctors: 113 images	Sensitivity: 78.7%; Specificity: 94.7%; Accuracy: 91.5%	Limited application and no clinical comparison	75
PU	CNN	Patches, pixels and color and textural features	Igurko Hospital and National Pressure Ulcer Advisory Panel: 22 images	Overall average classification accuracy: 92.01%	directly extrapolated to other burn wounds or skin tumor	76
DFU	A pre-trained DNN for feature extraction and classification at the patch-level	Patchs and pixels	350 images	Accuracy than 80%	Fewer training cases	77
Infection/inflammation,PU, Burn, Trauma, DFU	U-Net with EfficientNet and U-Net with MobileNetV2	Standard augmentations of images and pixels; Incorporates automatic color and measurement calibration	Photos from smart phone: 31 wound images	IoU: 0.6964	Small dataset and the imbalance classes	78
DFU	DNN (MobileNetV1 model)	Pixels (plus three color channels)	Wound Care Center of Christliches Klinikum Melle Germany: 326 augmented images	Precision: 0.67; Accuracy: 0.69.	Automatic wound documentation; Validation statistics should be further improved	80
DFU	State-of-the-art DNN for semantic segmentation fully CN: Seg Net, Unet, FCN8, FCN 16 and FCN 32	Patches and pixels, a set of color and textural features	Hospital Nacional Dos de Mayo and CHRO Hospital: 219 images	Accuracy: 92.68%; DICE index: 75.74%	High robustness especially for slough and GT; Fewer training cases	81
DFU	A conventional DL technique for Superpixel Colour Descriptor	Standard augmentations of images and Superpixels	628 cases infection and 831 cases non-infection	AUC: 0.73	Difficult to distinguish between ischemia and infection in machine vision	82
Chronic wound	A fuzzy divergence based thresholding by minimizing edge ambiguity and statistical learning algorithms; Bayesian classification and SVM	A set of color and textural features; Hue, saturation, and intensity	CW images from Medetec medical image database: 74 wound images	Accuracies (GT: 86.94%, slough: 90.47%, NT: 75.53%) Overall accuracy: 87.61%	Limited application; Few features	83
Complex wounds with five categories	Naive Bayes, Logistic Regression, and Random Forest	A pixel encodes (RGB intensity)	27 images acquired from 10 patients	FI of Random Forest: 0.9718	High precision; Few mages	84
Wound	Multi-label CNN ensemble, Deepwound,	Image pixels and corresponding labels	1335 smartphone wound images	AUC than 0.81	Don't consider blur detection	85
Murine wound	U-Net segmentation network	H&E skin sections	863 cropped wound images	Accuracy ≥90%	murine skin tissue	86

diabetic foot ulcers: DFU; venous ulcers: VU; pressure ulcers: PU; machine learning: ML; Deep learning: DL; area under the curve: AUC; support vector machine: SVM; convolutional neural networks: CNN; deep neural network: DNN; deep convolutional neural networks: DCNN; regions of interest: ROI; k-nearest neighbors: KNN; fully convolutional network: FCN; granulation tissue: GT; necrotic tissue: NT.

**Table 5:** The application, advantages, and limitations of different AI models and algorithms in wound repair

Model		Algorithms	Application	Advantage	Limitation	Extensibility	Ref.
Shallow- ML	SVM		Wound type classification; Wound area and depth measurement; Predict of wound healing	High global performance rates; Generalize the difficult wound segmentation	Accuracy limited by conditions of image capture; Can't produce fine contour	Integrate into smart phone; Easily extended to higher dimensions	24, 65, 102
	SVM with 3rd order polynomial kernel		Wound area measurement	Higher accuracy than Bayesian classifier	Insufficient sample	Diagnose skin tumours and other skin lesions	83
	K-means		Wound type classification; Predict of wound healing	Assess tissue healing in terms of granulation categories; Efficiently analyze the wound healing status	Lack the ability to detect all granulation regions; Complication of image capture process	Home care	103
	Random forest		Wound type classification; Wound area measurement	Enough classifying speed; High accuracy compared with SVM	Insufficient sample	Integrate into mobile device	26, 84, 166
	Classification tree		Predict of wound healing	Simple algorithm; Strong interactions between predictor variables	Predictive power relies on interactions between predictor variables	Applies to a broad range of wound etiologies	111, 112
DL	GoogLeNet		Wound area measurement	Overcome the differences in the computer vision perspective	A light-weight framework	Classify the other skin lesions	23
	MobileNet		Wound type classification; Tissue type classification	Automatic wound documentation; Explainable AI model	Validation statistics should be further improved	-	28, 78, 80
	ConvNet		Wound type classification; Predict of wound healing	Distinguish between wounds that look very similar; Amputation wound healing with a high degree of accuracy	Large consumption of computing resources	Classify the other skin injury	109, 110
	FCN		Wound type classification; Tissue type classification; Wound depth measurement	Own dataset with more wound images; Replace empirical and imprecise manual measurement	Draw irregular boundaries; Instead of learning from human experts; Class imbalance	Classify the other skin lesions Predict key factors influencing healing; Integrate into resource-constrained platform devices	46, 69, 81, 105
	Mask R-CNN		Wound tissue type classification	Classification performance can be comparable to clinical; Images of DFU without performing any preprocessing	Unable to classify burn depths on segmentation; No comparison with other frameworks; Real-time performance is poor	-	43, 48, 49
	U-Net		Wound type classification; Tissue type classification; Wound depth measurement	Appropriately supervised data to improve accuracy; Achieve accurate DFU tissue classification with small samples	Race bound; Not consider 3D structures; Fewer training images and good healing result	Diagnose skin tumours and other skin lesions; Integrate into mobile device	23, 44, 53, 68, 78, 80, 86
	VGG16		Wound area measurement	Higher precision than the traditional chronic wound image	Insufficient sample	Home care	44, 49
	ResNet		Wound type classification; Wound area and depth measurement	Quickly diagnose of patients with burn higher precision than VGG-16, GoogleNet	Insufficient sample; Fewer training images and final healing result	-	26, 35, 43, 66, 68
	Bilinear CNN (Bi-CNN)		Tissue type classification	Higher precision than AlexNet, VGG16, ResNet, Densenet	Class imbalance	Other medical image classification	70
	DnCNN		Predict of wound progress	Accelerate training and enhance denoising outcomes; Precisely capture nerve fibers	Not elucidate the morphological characteristics of cutaneous nerves in 3D; Real-time performance is poor	Neural prediction of other tissues; Precision medicine	108
Others	Multialgorithm (Neural networks and Bayesian classifiers, etc.)		Tissue type classification; Wound area and depth measurement; Predict of wound progress; Personalized treatment	High global classification accuracy rates; Well-adapted; Increased robustness	Increase in complexity; Need more data types	Classify the other skin injury; More clinical setting	52, 65, 75, 97, 106, 118
	AI chatbot (IDX-DR, ChatGPT, etc.)		Personalized treatment and lifestyle recommendations; Predict of wound healing	Generate tailored recommendations based on the patient's specific needs; Language comprehension and generation	limited by the information it has within its history plus the information it is given in a particular scenario	Complex wound care; Diverse health care settings; patient education	120, 139.
	Mobile application (Burn Med, C4W, Swift, TA, etc.)		Wound area and depth measurement; Predict of wound progress; Personalized treatment	High accuracy; Faster detection of high risk wounds	Accuracy depends on camera; Captured photos are prone to color differences	More clinical setting; Telemedicine; Home monitoring	56, 62, 103, 118, 119
							

Artificial intelligence: AI; support vector machine: SVM; machine learning: fully convolutional network: FCN; CNN; deep neural networks; ML, deep learning: DL; diabetic foot ulcers: DFU; 3D: three-dimensional.

## Abbreviations

AI: Artificial intelligence
ML: Machine learning
NN: Neural networks, DL: Deep learning
HER: Electronic health records
AUC: Area under the curve
SVM: Support vector machine
CNN: Convolutional neural networks
DNN: Deep neural network
DCNN: Deep convolutional neural networks
ROI: Regions of interest
RCNN: Regional convolutional neural network
R101FA: Residual network-101 with atrous convolution in feature pyramid network
R101A: Residual network-101 with atrous convolution
KNN: K-nearest neighbors
DANN: Deep artificial neural networks
DnCNN: Denoising convolutional neural network
FCN: Fully convolutional network
YOLO: You only look once
DC: Dice coefficient
IoU: Intersection over union
DWMS: Digital wound measurement system
ICC: Intraclass correlation coefficient
DFU: Diabetic foot ulcers
AU: Arterial ulcers
VU: Venous ulcers
PU: Pressure ulcers
VLU: Venous leg ulcers
CAD: Computer-aided diagnosis
ICD: Intelligent cell detection
GT: Granulation tissue
ET: Epithelial tissue
NT: Necrotic tissue
3D: Three-dimensional
NNC: Neural network controller
IHC: Immunohistochemical
LIC: Linear iterative clustering
WBP: Wound bed preparation
TSA: Trichostatin A
VOC: Volatile organic compounds
AMPs: Antimicrobial peptides
HDAC4: histone deacetylase 4
GDPR: General Data Protection Regulation
HIPAA: Health Insurance Portability and Accountability Act
NLP: Natural language processing
